# What Do Ethical Guidelines for Epidemiology Say About an Ethics Review? A Qualitative Systematic Review

**DOI:** 10.1007/s11948-016-9829-3

**Published:** 2016-11-15

**Authors:** Jan Piasecki, Marcin Waligora, Vilius Dranseika

**Affiliations:** 1 REMEDY, Research Ethics in Medicine Study Group, Department of Philosophy and Bioethics, Faculty of Health Sciences, Jagiellonian University, Medical College, Michalowskiego 12, 31-126 Krakow, Poland; 2 Department of Logic and History of Philosophy, Vilnius University, Vilnius, Lithuania

**Keywords:** Ethics of epidemiological research, Multicenter studies, An ethics review in epidemiology, Institutional Review Board, Research Ethics Committee, Ethics of public health studies, Qualitative review, Systematic review

## Abstract

Epidemiological research is subject to an ethics review. The aim of this qualitative review is to compare existing ethical guidelines in English for epidemiological research and public health practice in regard to the scope and matter of an ethics review. Authors systematically searched PubMed, Google Scholar and Google Search for ethical guidelines. Qualitative analysis (constant comparative method) was applied to categorize important aspects of the an ethics review process. Eight ethical guidelines in English for epidemiological research were retrieved. Five main categories that are relevant to the review of epidemiological research by Institutional Review Boards/Research Ethics Committees were distinguished. Within the scope of main categories, fifty-nine subcategories were analyzed. There are important differences between the guidelines in terms of the scope and matter of an ethics review. Not all guidelines encompass all identified ethically important issues, and some do not define precisely the scope and matter of an ethics review, leaving much to the ethics of the individual researchers and the discretion of IRBs/RECs.

## Introduction

An epidemiologist writing a research proposal must keep in mind not only scientific, but also ethical principles for research involving human subjects. The researcher can be especially interested in the necessity of submitting her proposal for an ethics review to an Institutional Review Board (IRB) or Research Ethics Committees (REC). She might be concerned about the scope and the matter of an ethics review. For instance, she could wonder, whether the study using unidentifiable samples from medical practice needs to be a subject of an ethics review (the scope) or what standards of informed consent forms would be applied by an IRB/REC. There are several national and international documents that have been developed since the 1980s, exclusively guiding the conduct of epidemiological studies. These guidelines can inform the epidemiologist about ethical principles, standards and procedures (McKeown et al. 2003; International Society for Environmental Epidemiology 2012; International Epidemiological Association 2007; Council for International Organizations of Medical Sciences 2008; Soskolne and Light 1996; Nakayama et al. 2005). The main goal of our research was to compare existing guidelines for epidemiological and public health research in regard to the scope and matter of an ethics review. These ethical documents are important not only to researchers, but also may be used by members of an IRB or REC conducting an ethics review, as well as by policymakers delineating national or international regulations. Therefore, gaining a more comprehensive picture of existing guidelines, realizing similarities and differences between the scope and the matter of an ethics review, seem to have both theoretical and practical significance. The results of this systematic study can be a point of departure for future discussion over the scope and matter of an ethics review. Finally, the results can contribute to the development of new, revised versions of ethics guidelines for epidemiological and public health studies. In this article we present the methods and results of our review, as well as discussion of some guideline comparisons.

## Methods

### Search Strategy

We constructed a search methodology to determine sample guidelines that would meet three conditions: transparency, replicability and lack of bias. We identified the guidelines through a systematic search in PubMed and searches in Google Scholar and Google Search. In the PubMed search, we used the following combination of terms: (code OR guideline OR codes OR guidelines) AND (epidemiology OR “public health”) AND ethics AND research. In Google Scholar and in Google Search, we used a combination of key terms: epidemiological research, epidemiology, public health, ethics and ethical guidelines. Because of the vast number of search results in both Google Scholar and Google Search, we limited our search to the first 300 hits sorted by relevance.

### Inclusion/Exclusion Criteria

We wanted to retrieve a sample of guidelines that could be used for epidemiological or public health research either by researchers or by members of IRBs/RECs. Inclusion criteria were met when guidelines explicitly introduce themselves as ethical guidelines regulating epidemiological or public health research and when they contain at least one paragraph defining the scope and matter of an ethics review. Inclusion criteria were not met when guidelines were general, regulating all kinds of human research, even if they had a section devoted to epidemiological research. Guidelines regulating only one, specific type of epidemiological research were excluded as well. By employing these criteria, we intended to avoid two kinds of interpretation problems: specification and generalization. General guidelines and guidelines containing an epidemiological section might require interpretation to identify, whether they concern, for instance, all types of multicenter studies or only multicenter clinical trials. Guidelines regulating a specific kind of epidemiological research would require additional interpretation to determine whether a certain provision has universal validity or it has application only in a specific context. Also articles, reviews, letters, books, editorials, dissertations, and notes were excluded from the analysis. All guidelines which were not published in English were excluded as well.

### Extraction of Guidelines Sample

During the search in PubMed, two authors (JP, MW) independently screened titles and got samples of documents that concern ethical issues in epidemiology and public health. In the next step, one author (JP) screened the contents of these articles to identify guidelines. In two consecutive searches in Google Scholar and in Google Search, two authors (JP and MW) independently screened first 300 titles and abstracts of both searches to identify additional documents. These searches resulted in a sample of documents and all three authors had to agree, whether they met inclusion/exclusion criteria. In case of disagreement, authors reached consensus by discussion.

### Data Extraction and Qualitative Analysis

We used the constant comparative method (CCM) to obtain all of ethical aspects concerning an ethics review within the analyzed guidelines and to build a grid of categories. The main goal of this research activity is descriptive, and extraction of the categories has a generally inductive character. The CCM is a qualitative methodology that consists of a close reading and re-reading of text and then coding/tagging subsequent passages of text with categories that encompass the meaning conveyed by the text (Gibbs 2008; Rapley 2008; Boeije 2002; Dye et al. 2000; Glaser 1965). The result of CMM is a grid of categories. Each category is a general tag that conveys general meaning, encompassing individual wording of many analyzed texts. In the first step, we extracted all passages that referred to an ethics review of epidemiological studies. The extraction was done independently by all authors (JP extracted all 8 guidelines, MW and VD extracted 4 each; thus, each guideline was analyzed twice, by two separate coders). JP constructed the main grid of categories using as a point of departure the extracted material and applying the CCM. The grid that was later checked and discussed with MW and VD to minimize the subjective character of categorization. All authors’ background is philosophy and bioethics, particularly research ethics. In bioethics similar approaches have been already described in the literature (Hirschberg et al. 2014; Strech et al. 2013; Henderson et al. 2013).

## Results

### Search and Selection

**Fig. 1:**
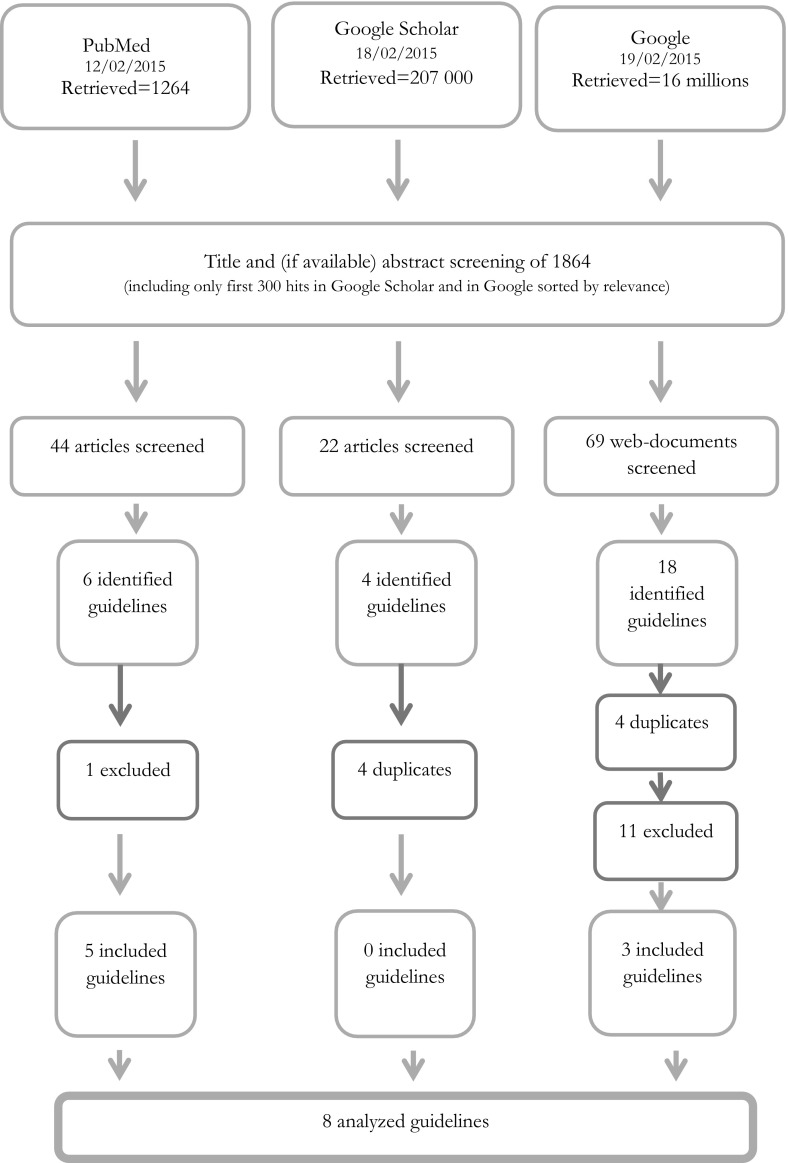
Results of searches in databases

During the search in PubMed 1264 documents were retrieved. Screening of titles resulted in a sample of 44 documents that concern ethical issues in epidemiology and public health. This set of documents informed us about 6 different guidelines. One document was excluded because the guidelines did not contain a paragraph defining a scope and matter of an ethics review (see Fig. 1). Therefore 5 guidelines were included for further analysis. In Google Scholar, we got an abundance of hits (around 207,000). Therefore, we limited our screening to the first 300 hits sorted by relevance. After this step, we got a list of 22 documents that allowed us to identify 4 guidelines: none of the 4 were new. Therefore, we did not retrieve additional guidelines. In the third search through Google Search (screening limited to first 300 hits), authors (JP and MW) independently retrieved 69 web-documents that were fully screened. Eighteen guidelines were obtained, but only three were incorporated to the sample, others either did not meet inclusion criteria (11 documents, see Fig. 1) or had been previously identified (4 documents, see Fig. 1). Excluded guidelines did not meet one or two of the inclusion criteria: six of them did not define the scope and matter of an ethics review. Three were considered to be too narrow. And three were considered too broad, one of them also did not define the scope and matter of an ethics review. Summing up, we retrieved and finally analyzed 8 documents. Figure 1 presents the consecutive steps of our search and Table 1 contains included and analyzed documents.

**Table 1:** The list of guidelines included to qualitative analysis

Title	Organization	Year of latest version
*Included and analysed documents*		
Ethical Guidelines for Epidemiologists (IEF-EGE)	Industrial Epidemiology Forum	1991
American College of Epidemiology Ethics Guidelines (ACEEG)	Amercian College of Epidemiology	2000
Good Epidemiological Practice. IEA Guidelines for proper conduct in epidemiological research (IEA-GEP)	International Epidemiological Association	2007
Guidelines for Good Phamacoepidemiology Practices (GGPP)	International Society for Pharmacoepidemiology	2007
International Guidelines for Epidemiological Studies (CIOMS-IEGES)	Council for International Organizations of Medical Sciences, World Health Organization	2008
Ethical Guidelines for Epidemiological Research (JAPAN-EGES)	Ministry of Education, Culture, Sports, Science and Technology, Ministry of Labour and Welfare, Japan	2008
Ethical Guidelines for Observational Studies: Observational research, audits, and related activities. Revised edition (NEW-ZEALAND-EGOS)	National Ethics Advisory Committee, Ministry of Health, New Zealand	2012
Ethical Guidelines for Environmental Epidemiologists (EGEE)	International Society for Environmental Epidemiology	2012

### Qualitative Analysis

We identified five main categories in the guidelines that define the IRBs/RECs’ role in epidemiological research. These categories are information policy, protection of subjects, guards for research integrity, formal and operational requirements, type of studies (that are either reviewed or exempt from review). In each category, we distinguished a set of subcategories that describe the specificity of IRBs/RECs’ responsibilities. There are fifty-nine subcategories that are relevant to an ethics review of epidemiological studies. The full list of categories and subcategories is presented in Table 2.

**Table 2:** The scope and matter of an ethics review in epidemiological studies

Symbol	Name	Title of guidelines				
		Ethical Guidelines for Epidemiologists (IEF-EGE)	American College of Epidemiology Ethics Guidelines (ACEEG)	Good Epidemiological Practice. IEA Guidelines for proper conduct in epidemiological research (IEA-GEP)	Guidelines for Good Phamacoepidemiology Practices (GGPP)	International Guidelines for Epidemiological Studies (CIOMS-IEGES)
*A. Information policy*						
A1	Scope/content/procedures of obtaining informed consent	X		X		X
A2	Scope/content/procedures of obtaining proxy consent					X
A3	Scope/content/procedures of obtaining assent/agreement of incapable subjects					X
A4	Deviation from standard written informed consent form	X	X			X
A5	Withholding information, deception of the subject, debriefing	X				X
A6	Access to information (including clinical and personal data) collected in the course of study					X
A7	Communication with subjects, who did not give their IC			X		X
A8	Communication with community; community consultation and involvement					X
A9	Communication of results to media and general public	X				X
A10	Communication of results in scientific journals					X
*B. Protection of subjects*						
B1	Subjects’ rights, well-being, and safety	X	X			X
B2	Proper balance between risk and benefits for subjects and the public	X		X		X
B3	Report of adverse events (physical and/or psychological)		X			
B4	Equitable enrollment of subjects	X				X
B5	Use of placebo/no-treatment					X
B6	Exceptional breach of confidentiality		X			
B7	Waiver of informed consent (also in research utilizing existing personal information/specimen)	X	X	X		X
B8	Procedures protecting subjects’ privacy and confidentiality	X	X			X
B9	Collecting, use, reuse, sharing exchanging, and final destination of data (personal information and/or biospecimens, genetic information)	X		X		X
B10	Anonymization of data/biospecimens					X
B11	Request for removal of data from the study					X
B12	Safeguards protecting vulnerable subjects	X		X		X
B13	Payment, reimbursement, medical services in the study, compensation					X
B14	Plans for studies in emergency					X
B15	IRB/REC from sponsoring country reviews research protocol in host country					X
B16	IRB/REC from host country ensures that all ethical provisions are met and study is in accordance with local values					X
*C. Guards for research integrity *						
C1	All ethical aspects of the study	X	X			X
C2	Scientific merit of the study	X				X
C3	Oversight of the study conduct		X	X		X
C4	Significance of observational character of most epidemiological studies			X		X
C5	Improving ethical/scientific/operational aspects of studies	X				X
C6	Conflict of interests			X		X
C7	Ethical approval	X	X			X
C8	Reporting unethical behavior and/or misconduct					X
*D. Formal and operational requirements*						
D1	Independence		X			X
D2	Rules of proceeding	X			X	X
D3	Communication of proceeding rules					X
D4	Obstruction of the study			X		X
D5	Disclosure and avoidance of conflict of interest			X		X
D6	Adequate composition of membership	X				X
D7	Periodical replacement of members					X
D8	Respecting sponsors’ rights			X		X
D9	Different levels of functioning (local/national/international)		X			X
D10	Appeal procedure	X				
D11	Fast-track review	X			X	
D12	Cooperation between IRBs/RECs	X				X
*E. Types of studies*						
E1	Studies involving human beings	X	X		X	X
E2	Use of identifiable data without informed consent		X			X
E3	Use of human specimens without informed consent		X	X		X
E4	Studies of unclear nature					X
E5	Sensitive topics studies					X
E6	Studies using clinical data without informed consent		X			X
E7	Studies using publicly available data					X
E8	Minimal risk		X			X
E9	Studies using anonymized data/specimens				X	X
E10	Studies without informed consent regulated by local laws				X	X
E11	Public health, routine surveillance		X			X
E12	Studies in emergency, acute communicable diseases		X			X
E13	Simple aggregation of records					
Numbers of categories mentioned in the guidelines		21	18	13	5	55

Symbol	Name	Title of guidelines			
		Ethical Guidelines for Epidemiological Research (JAPAN-EGES)	Ethical Guidelines for Observational Studies: Observational research, audits, and related activities. Revised edition (NEW-ZEALAND-EGOS)	Ethical Guidelines for Environmental Epidemiologists (EGEE)	Number of guidelines mentioning the category
*A. Information policy*					
A1	Scope/content/procedures of obtaining informed consent	X	X	X	6
A2	Scope/content/procedures of obtaining proxy consent	X			2
A3	Scope/content/procedures of obtaining assent/agreement of incapable subjects	X			2
A4	Deviation from standard written informed consent form	X		X	5
A5	Withholding information, deception of the subject, debriefing				2
A6	Access to information (including clinical and personal data) collected in the course of study				1
A7	Communication with subjects, who did not give their IC	X			3
A8	Communication with community; community consultation and involvement	X		X	3
A9	Communication of results to media and general public			X	3
A10	Communication of results in scientific journals				1
*B. Protection of subjects*					
B1	Subjects’ rights, well-being, and safety	X	X	X	6
B2	Proper balance between risk and benefits for subjects and the public	X	X		5
B3	Report of adverse events (physical and/or psychological)				1
B4	Equitable enrollment of subjects	X			3
B5	Use of placebo/no-treatment				1
B6	Exceptional breach of confidentiality			X	2
B7	Waiver of informed consent (also in research utilizing existing personal information/specimen)	X	X	X	7
B8	Procedures protecting subjects’ privacy and confidentiality	X	X	X	6
B9	Collecting, use, reuse, sharing exchanging, and final destination of data (personal information and/or biospecimens, genetic information)	X	X	X	6
B10	Anonymization of data/biospecimens				1
B11	Request for removal of data from the study				1
B12	Safeguards protecting vulnerable subjects	X		X	5
B13	Payment, reimbursement, medical services in the study, compensation	X			2
B14	Plans for studies in emergency				1
B15	IRB/REC from sponsoring country reviews research protocol in host country	X			2
B16	IRB/REC from host country ensures that all ethical provisions are met and study is in accordance with local values			X	2
*C. Guards for research integrity*					
C1	All ethical aspects of the study	X		X	5
C2	Scientific merit of the study	X		X	4
C3	Oversight of the study conduct	X		X	5
C4	Significance of observational character of most epidemiological studies				2
C5	Improving ethical/scientific/operational aspects of studies			X	3
C6	Conflict of interests	X	X	X	5
C7	Ethical approval	X		X	5
C8	Reporting unethical behavior and/or misconduct				1
*D. Formal and operational requirements*					
D1	Independence				2
D2	Rules of proceeding	X		X	5
D3	Communication of proceeding rules	X		X	3
D4	Obstruction of the study			X	3
D5	Disclosure and avoidance of conflict of interest	X		X	4
D6	Adequate composition of membership	X		X	4
D7	Periodical replacement of members				1
D8	Respecting sponsors’ rights	X			3
D9	Different levels of functioning (local/national/international)	X			3
D10	Appeal procedure				1
D11	Fast-track review	X		X	4
D12	Cooperation between IRBs/RECs	X		X	4
*E. Types of studies*					
E1	Studies involving human beings			X	5
E2	Use of identifiable data without informed consent	X	X	X	5
E3	Use of human specimens without informed consent	X	X	X	6
E4	Studies of unclear nature				1
E5	Sensitive topics studies				1
E6	Studies using clinical data without informed consent			X	3
E7	Studies using publicly available data				1
E8	Minimal risk	X	X		4
E9	Studies using anonymized data/specimens	X			3
E10	Studies without informed consent regulated by local laws	X	X		4
E11	Public health, routine surveillance	X	X		4
E12	Studies in emergency, acute communicable diseases	X			3
E13	Simple aggregation of records	X			1
Numbers of categories mentioned in the guidelines		36	12	28	

#### Information Policy

IRBs/RECs have authority to review all kinds of information concerning research project, and they assess, how researchers fulfill their obligation to “disclose information”. In epidemiological studies, the duty to inform is not limited to obtaining informed consent. It is not always feasible to inform all subjects, but nevertheless, researchers may have a duty to inform general public and give a participant an opportunity to withdraw from the study. Moreover, epidemiologists, as well as public health workers, have a duty to communities to disclose information about public health threats and major determinants of health and causes of disease. The duty to disclose information covers also communication of study results to the scientific community. Some of the analyzed guidelines require an ethics review of all these aspects of communication with the public and subjects, other limit the scope of an ethics review to some of these issues or even only to the requirement of informed consent.

#### Protection of Subjects

The second category is “Protection of subjects” and it encompasses all provisions that are envisaged for the protection of study participants. One of the key roles of IRBs/RECs is to ensure that the well-being of participants is not subject to unjustified risk. Some guidelines give IRBs/RECs an important role in protecting privacy and confidentiality, stating that an IRB/REC has to approve all exceptional breaches of confidentiality. Also, most guidelines bestow upon IRBs/RECs a power to decide when and if the requirement to obtain informed consent from the study participants might be waived. There are two different subcategories that refer to informed consent. The first subcategory, “Scope/Content/Procedures of Obtaining informed consent,” is under the broader category “Information policy”; the second, “Waiver of informed consent,” falls under “Protection of subjects”. These two categories are thought to reflect two different aspects of the informed consent requirement. On the one hand, the requirement of informed consent refers to the duty to disclose information. On the other hand, obtaining informed consent is thought to be an instrument protecting the subject’s best interests.

#### Guards for Research Integrity

The third category, “Guards for research integrity,” refers to powers of IRBs/RECs to guard a study from scientific misconduct. An IRB/REC issues its opinion on scientific and ethical merit of a study and monitors the possible or actual conflict of interests. Also, some guidelines give IRBs/RECs responsibility to report to the authorities the unethical and unlawful behavior of scientists.

#### Formal and Operational Requirements

The fourth category, “Formal and operational requirements,” contains all important aspects of the institutional functioning of IRBs/RECs. The efficient and ethical work of an IRB/REC requires it to be equipped not only with authority or powers, but also to have adequate administrative procedures in place. IRBs/RECs have to set and announce their rules of proceeding. Members of an IRB/REC are required to have proper competences, which may differ between different members. IRB/REC members also should disclose and avoid conflict of interests.

#### Type of Studies

According to the guidelines not all epidemiological or public health studies require an ethics review. For instance, according to most guidelines public health surveillance conducted by a governmental institution is not required to be reviewed by an IRB/REC, even if such monitoring uses identifiable data. Many guidelines, however, require an ethics review when identifiable data is being used. The category “Types of studies” summarizes criteria for either necessity of an ethics review or for exemption from the review process.

### Similarities/Differences

An important feature of the set of documents is it’s heterogeneity. The guidelines differ with regards to length, scope, form and purpose. The guidelines are issued by different organizations and are intended to serve different goals. None of the subcategories that we distinguished appear in all guidelines. Only one subcategory (B7—waiver of informed consent) appears in seven guidelines. The next most frequent categories that appear in six different guidelines are A1 (scope/content/procedures of informed consent), B1 (subjects’ rights and well-being), B8 (procedures protecting subjects’ privacy and confidentiality), B9 (collecting, use, reuse, sharing, exchanging and final destination of data), and E3 (use of human specimen without informed consent). Subcategories A4 (deviation from the standard written informed consent form), B2 (proper balance between risk and benefits for subjects and public), B12 (safeguards protecting vulnerable subjects), C1 (all ethical aspects of the study), C3 (oversight of the study conduct), C6 (conflict of interests), C7 (ethical approval), D2 (rules of proceeding), E1 (studies involving human beings) and E2 (use of identifiable data without informed consent) appears in five documents.

#### How Should IRBs/RECs Protect Participants?

The main task of IRBs/RECs is usually defined as protection of participants’ rights and interests (B1) and the fair distribution of risk and benefits (B2). In most of the guidelines, one can find provisions that an IRB/REC has a responsibility to oversee procedures and means of protecting confidentiality and privacy of research participants (B8). Guidelines usually stipulate that personal data should be physically and electronically protected properly. Some, for instance JAPAN-EGES, give a very detailed description of protection measures: systematic, human, physical, and technological. This also implies that IRBs/RECs should have expertise in reviewing such procedures and technologies (D6).

#### What Information has to be Given to Subjects?

Participants have a right to be properly informed about research. One of the basic responsibilities of an IRB/REC is to monitor informed consent procedures (A1). But this obligation is differently described in the guidelines. In most guidelines, one can find only general provisions that an IRB/REC should review or/and monitor informed consent forms and procedures. But, for instance, CIOMS-IEGES and JAPAN-EGES explicitly list the information that must be provided to research participants. The CIOMS-IEGES list is intended to exhaust probably all possible variants and leave to the IRB/REC’s discretion which elements from the list should be included in a particular informed consent form. Moreover in some circumstances subjects cannot be fully informed about the very nature of the study; in other cases, the IRB/REC might even allow for the deception of subjects (A5). Only two guidelines consider this possibility and give the IRB/REC authority to judge whether such an instrument is ethically and scientifically justified (CIOMS-IEGES, IEF-EGE).

Almost all guidelines refer to the process of communication with the public; nevertheless, not all give an IRB/REC an authority to assess the plan of communication (A8). An IRB/REC might approve the method and timing of communication of results (A9). For instance, according to EGEE “Studies in progress should not report results to the media without prior authorization by a properly constituted IRB/REB (International Society for Environmental Epidemiology 2012).” IEA-GEP contain a whole paragraph devoted to media communication of results, but do not mention the necessity for IRB/REC review (International Epidemiological Association 2007). Authority of IRBs/RECs may embrace as well a plan of publication of multicenter research. According to CIOMS-IEGES, individual researchers should not independently publish the results and the data should be analyzed by the research steering committee (Council for International Organizations of Medical Sciences 2008).

#### How Should IRBs/RECs Operate?

Streamlining of the review procedure is essential for the speed and efficacy of research. Cooperation between IRBs/RECs is allowed by different guidelines. Nevertheless local IRBs/RECs might have the authority to “prevent a study that they believe to be unethical” (Council for International Organizations of Medical Sciences 2008). In addition, local IRBs/RECs are given the same task to “protect the research subjects” (Council for International Organizations of Medical Sciences 2008).

The issue of unnecessary delay to the instigation of the research due to inflexible an ethics review is directly addressed both in IEA-GEP and in CIOMS-IEGES (C3, D4, D11). Also EGEE guidelines point out that the process of review might unduly slow down the study (International Society for Environmental Epidemiology 2012). There is a common opinion that IRBs/RECs should not apply the same standards to more risky interventional research and relatively safe observational studies. The system of IRB/REC review was originally designed for regional and local studies. Therefore, it poses a challenge for epidemiological studies, which cover large populations in many sites.

#### What Kinds of Studies Should Lie Within the Scope of the Irbs/Recs Review?

Most guidelines require ethical approval of all research involving humans (C7), although the same guidelines in certain cases allow exemption from review. Usually studies that do not require an ethics review are those based on simple aggregation of already existing records (E13) or use of administrative databases and records without personal identifiers (E9). Also routine public health surveillance or research in states of emergency on a societal scale (e.g. an epidemic) are exempted from an ethics review (E11). In other types of studies, especially in cases when researchers use biospecimens and identifiable records without informed consent, an ethics review is explicitly required. Our analysis therefore shows that in most guidelines there are two independent conditions for an ethics review in epidemiological studies. The first is involvement of human beings. If a study involves human beings, it should be approved by an IRB/REC. CIOMS-IEGES determine that research involves human beings when either the investigator directly obtains information from individuals and groups, or otherwise acquires identifiable *private* information. Other guidelines either do not contain more precise definition of studies involving humans or do not give such a definition at all (e.g. IEA-GEP). The second condition is the classification of a researchers’ activity. A researcher might either conduct biomedical research or practice public health. Public health practice does not require an ethics review, in contrast to epidemiological research (E11). Nevertheless, the borderline between research and practice in epidemiology and public health is blurred. Public health practice is associated with gathering information and production of generalizable knowledge and in many cases it poses the same risks as research (Willison et al. 2014). The guidelines do not provide a conceptual distinction between research and practice. The difference between research and public health practice has rather a legal and institutional than essential character (E10). Some suggest that in case of doubt, whether a certain activity constitutes research or practice the question should be answered by an IRB/REC (McKeown and Leaner 2009).

## Discussion

### Limitations

Our study might have some limitations. One can criticize the composition of the sample, saying, for instance that ethical guidelines should be searched through well-known organizations and widely recognized handbooks for epidemiological ethics. We did not decide on this strategy because our goal was to create a transparent, replicable and unbiased search methodology. Although before we conducted a systematic search in the databases, we had unsystematically searched the literature and we had identified the most recognizable ethical guidelines for epidemiological research. Therefore our systematic search has been somehow verified by the previous, preliminary research. We also purposively limited our analysis to guidelines published in English, and that might have limited the scope of distinguished categories. Another problem might be the subjectivity of inducted categories and subcategories. This problem was addressed by having two or three separate researchers at each level of data analysis or synthesis. We believe that taking into account mentioned limitations, our research remains valid and reliable in drafting a grid of issues important in an ethics review of epidemiological studies.

### Ethically Significant Matters

Our analysis suggests that there are some aspects of epidemiological studies that, according to most of the guidelines, lie within the scope of an ethics review. It seems that most guidelines require IRBs/RECs to review either the most important aspects of the study or issues that are specific for epidemiological studies. Process, procedure and content of informed consent, balance between benefits and risk, as well as oversight of conflict of interests belong to the first mentioned category. Whereas, ethical oversight of procedures protecting a subjects’ privacy and confidentiality, use, reuse, sharing exchanging, and final destination of personal data and biospecimen belong to the second category.

On the other side are categories that are mentioned only by one or two guidelines. There are two types of these rarely mentioned categories. The first type covers specific aspects of the study design, that can be virtually present in more general provision of the other guidelines. For instance, CIOMS-IEGES require an ethics review in studies on sensitive topics. But this kind of study is a subcategory of studies involving human beings that, according to most guidelines, should be subject to an ethics review. The second type, and it seems the most important, are ethically important provisions, sometimes also specific for epidemiological studies, that appear only in a few guidelines. This rare appearance of important categories might suggest either incompleteness or provisional character of less elaborated guidelines. For instance, only CIOMS-IEGES require IRBs/RECs to review access to information (including clinical and personal data) collected in the course of a study and allow for a possibility to request the removal of data from the study. Other guidelines do not mention such a possibility or leave it to the discretion of the director of the research institution (JAPAN-EGES).

It seems that access to personal data and the possibility of removal of personal information are crucial to protect a subjects’ autonomy. At the same time, finding personal data or removing it from large databases might pose a serious problem for researchers. Therefore, an independent IRB/REC should have the power to find a balance between the personal best interests and scientific merit of the study. Similarly, it seems that withholding information or even deception of subjects requires an ethics oversight. Some epidemiological studies cannot be conducted without withholding information from patients. For instance, a study concerning compliance with doctor’s instruction cannot be conducted, if patients are told the purpose of the study. We found many important aspects of an ethics review that are not mentioned in most guidelines. Therefore, it seems that some epidemiological guidelines are incomplete, provisional and may be used only as a supplement to more elaborate documents regulating research involving human beings.

### Burdensome Character of an Ethics Review

The burdensome character of the reviewing process for large, multicenter epidemiological and observational studies is well-known and described (Lux et al. 2000; Middle et al. 1995; Tully et al. 2000; Green et al. 2006; Thornquist et al. 2002; Greene and Geiger 2006). One may ask a question, whether differences between guidelines and use of not fully-developed ethics guidelines for epidemiological research contribute to this problem? Of course, the burdensome character of an ethics review might be ascribed to many different reasons. For instance, a system of review could be organized in an inappropriate way (Vaughan et al. 2012). Nevertheless, it seems that harmonization of guidelines, elaboration of documents that only superficially treat the issue of an ethics review, might have some impact on IRBs/RECs and their way of conducting an ethics review of epidemiological and public health studies.

## Conclusion

In this article we conducted a qualitative review of ethical guidelines for epidemiological and public health studies. Applying the constant comparative method, we obtained fifty-nine subcategories reflecting ethically important aspects of study design. We discovered important differences between guidelines in terms of the scope and matter of an ethics review. Not all guidelines encompass ethically important issues. Some did not define precisely the scope and matter of an ethics review, leaving much to the discretion of IRBs/RECs and ethics of researchers. Nevertheless, we also discovered some significant similarities among a majority of guidelines. Almost all analyzed documents require an ethics review of the ethically most fundamental aspects of all studies involving human beings (informed consent, conflict of interests) as well as issues specific to epidemiological research (safeguards for privacy). We hope that our findings can contribute to the discussion on an ethics review of epidemiological studies and help to harmonize guidelines and policies in the future.

## Abbreviations

ACEEG: American College of Epidemiology Guidelines (American College of Epidemiology 2000)
CCM: Constant Comparative Methods
GGPP: Guidelines for Pharmacoepidemiology Practices (International Society for Pharmacoepidemiology 2008)
EGEE: Ethical Guidelines for Environmental Epidemiologists (International Society for Environmental Epidemiology 2012)
CIOMS-IEGES: International Ethical Guidelines for Epidemiological Studies (Council for International Organizations of Medical Sciences 2008)
IEF-EGE: Industrial Epidemiology Forum, Ethical Guidelines for Epidemiology (Beauchamp et al. 1991)
IRB/REC: Institutional Review Board/Research Ethics Committee
JAPAN-EGES: Ethical Guidelines for Epidemiological Studies (Ministry of Education, Culture, Sports, Science and Technology, Ministry of Health, Labour and Welfare 2008)
IEA-GEP: Good Epidemiological Practice—IEA Guidelines for Proper Conduct in Epidemiological Research (International Epidemiological Association 2007)
NEW-ZEALAND-EGOS: Ethical Guidelines for Observational Studies. Observational Research, Audits and Related Activities (National Ethics Advisory Committee 2012)
